# MYC Participates in Lipopolysaccharide-Induced Sepsis via Promoting Cell Proliferation and Inhibiting Apoptosis

**DOI:** 10.22074/cellj.2020.6961

**Published:** 2020-09-08

**Authors:** Yin Li, Chengqi Kong, Lei Feng, Wenliang Tang, Mengwei Chen, Zhiyuan Zheng

**Affiliations:** 1. Emergency Department of Huadong Hospital, Fudan University, Yan’an Xi Road, Shanghai, China; 2. Cardiovascular Department of Huadong Hospital, Fudan University, Shanghai, China

**Keywords:** Cell Apoptosis, Cell Proliferation, Inflammation, Lipopolysaccharide, *MYC*, Sepsis

## Abstract

**Objective:**

This study aimed to explore the potential mechanism of *MYC* proto-oncogene, BHLH Transcription Factor (*MYC*) gene, on sepsis.

**Materials and Methods:**

In this experimental study, rat-derived H9C2 cardiomyocyte cells were cultured *in vitro*, followed by lipopolysaccharide (LPS) treatment with different concentration gradients. The cholecystokinin octapeptide (CCK-8) assay, enzyme-linked immunoassay (ELISA) assay, quantitative reverse transcription polymerase chain reaction (qRT-PCR), cell transfection, Western blot and flow cytometry were used to observe the cellular apoptosis and proliferation of cells in both treated LPS groups and normal control group.

**Results:**

The result of CCK-8 assay showed that silencing *MYC* inhibited cellular proliferation of sepsis in absence or presence of LPS treatment. ELISA assay showed that the expressions of tumor necrosis factor-α (TNF-α) and interleukin-6 (IL-6) were decreased in *MYC* silenced group, but they were increased after LPS treatment. Moreover, Flow cytometry assay showed that *MYC* silencing contributed to the apoptosis of sepsis cells. Furthermore, the expression of inflammatory factors showed that *MYC* silencing elevated the expression of inflammation factors.

**Conclusion:**

*MYC* might take part in the process of LPS induced sepsis through suppressing apoptosis and inducing cell proliferation. Moreover, *MYC* might reduce inflammation during the progression of LPS induced sepsis.

## Introduction

Sepsis is a kind of systemic inflammatory response syndrome (SIRS), caused by the infection (1). As the most common reason for the hospitalized death, the incidence of sepsis is 18 million cases per year worldwide (2). Poor organ function or insufficient blood flow is the outcome of severe sepsis in clinic (3). Although the influence factors and interventions for sepsis have been widely studied in both animal models and clinic (4, 5), details of the pathogenesis mechanisms are still unclear.

MYC proto-oncogene, BHLH Transcription Factor (*MYC*) are a group of early oncogenes including C-myc, N-myc and L-myc. The corresponding RNAs regulated by MYC participate in various biological functions including cell death, proliferation and mechanisms of drug resistance (6). *MYC* silencing by small interfering RNAs (siRNAs) revealed the importance of biological function of *MYC* in disease (7). Previous study showed that *MYC* signaling in inflammatory response can be used as a therapeutic target during the disease progression (8). Although sepsis is caused by the inflammatory immune response (9), detail of the function of *MYC* in this system is unknown. In our previous gene expression analysis, *MYC* were proved to participate in the pathogenesis of sepsis (10). Although these results provided a genomics information for sepsis progression, detail of the gene functions like *MYC* in pathogenesis of sepsis is yet unclear. Therefore, in the current study, we aimed to explore the potential function of *MYC* on sepsis progression through observing the differences of proliferation and apoptosis of cells in the treated lipopolysaccharide (LPS) groups and normal control group. This will provide new sight for gene-based target therapy of sepsis.

## Materials and Methods

### Cell culture and grouping

In this experimental study, rat-derived H9C2 cardiomyocyte cell line (Chinese Academy of Sciences, Shanghai, China) were cultured in Dulbecco’s Modified Eagle Medium (DMEM) culture medium (Gibco BRL, USA) containing 10% fetal bovine serum (FBS, Gibco BRL, USA) and 1% penicillin/streptomycin, followed by incubation at 37˚C with 5% CO_2_. Then, the H9C2 cells in logarithmic growth phase were digested with trypsin (0.25%, Gibco BRL, USA) and cultured in the 96-well plates (2×10^5 ^ cells/well, 37˚C, 5% CO_2_) for 24 hours.

When the cell density in plates was above 90%, they were divided into five groups including normal control (NC), LPS1 (treated with 1 μg/ml LPS), LPS10 (treated with 10 μg/ml LPS), LPS20 (treated with 20 μg/ml LPS) and LPS40 (treated with 40 μg/ml LPS) groups. Treatment time for all of these groups was 4, 8 and 24 hours, respectively.

### CCK-8 assay

A total of 5 mg/ml CCK solution (BIYUNTIAN Biotechnological Co., China) was used for the CCK-8 assay on H9C2 cells at 24, 48 and 72 hours. Subsequently, the plates were incubated at 37˚C with 5% CO_2_ for 24 hours and the absorbance at 450 nm was recorded by a microplate reader (Gene Co., Germany).

### ELISA assay

ELISA assay was performed to reveal the expression of MYC in each group. Briefly, interleukin-6 (IL-6) and tumor necrosis factor-α (TNF-α) levels were tested in the samples by ELISA kit (BioSource International, USA). Absorbance (OD) of each hole was measured at 450 nm wavelength in sequence by a microplate reader (Gene Co.).

### Quantitative reverse transcription-polymerase chain reaction assay

Total RNA extraction was performed using TRizol reagent (TaKaRa, Japan), and reverse transcribed using RevertAidTM First Strand cDNA Synthesis Kit (Thermo Fisher Scientific, USA). Quantitative reverse transcription-polymerase chain reaction (qRT-PCR) assay was performed on ABI7900FAST (Thermo Fisher Scientific, USA) and the primers were as follows:

*MYC*-

F: 5´-CCTCGCGTTATTTGAAGCCTG-3´

R: 5´-CACCGAGTCGTAGTCGAGGT-3´

*GAPDH*-

F: 5´-AGACAGCCGCATCTTCTTGT-3´

R: 5´-CTTGCCGTGGGTAGAGTCAT-3´.

PCR program was performed with thermocycling conditions: 50°C for 3 minutes, 95°C for 3 minutes, 40 cycles of 95°C for 10 seconds, 60°C for 30 second and melt curve of 60°C to 95°C (Increment 0.5°C for 10 seconds). The method of 2^-∆∆Ct^ (11) was used for the investigation of gene expression.

### Cell transfection

Specific shRNA sequence was designed and synthesized in the current study. Simply, shRNAs were inserted into the vector of pLKO.1-Puro at the restriction sites of AgeI and EcoRI. Then, the recombinant pLKO.1-Puro was transformed into DH5α competent cell, followed by the sequence identification of positive clones. The confirmed plasmid by DNA sequencing was extracted by CP6 adsorption column (Tiangen, China). The cells were transfected with plasmid carrying shRNA sequence by the lipofectamine 2000 (Thermo Fisher Scientific, USA). After 48 hours of transfection, the transfected cells were collected for further assay.

### Western blot

Total proteins of H9C2 were extracted by RIPA lysis buffer (BIYUNTIAN Biotechnological Co.). After centrifugation, the proteins were separated by SDSpolyacrylamide gel electrophoresis (10%), and transferred to the polyvinylidenefluoride membrane (Millipore, USA). 5% skimmed milk (0.75 g milk powder+15 ml PBS) was used for blocking the membrane for 1-2 hour(s) and incubated with primary antibodies (C-myc, rabbit monoclonal antibody, 57 kDa, 1:1000; Pax-2, rabbit monoclonal antibody, 45 kDa, 1:5000; GAPDH, rat monoclonal antibody, 36 kDa, 1:1000; Santa Cruz Biotechnology Inc., USA) overnight at 4°C. Then, the samples were treated with the secondary antibody (antirabbit, 1:10000; anti-rat, 1:5000; Cwbio, China) for 2 hours at 37°C. Protein brands were visualized with Millipore ECL Gel imaging system (Millipore, USA). Finally, the results were analyzed by TanonImage 4600 (Tanon, China).

### Flow cytometer

Apoptosis of the transfected cells was detected by flow cytometry. Briefly, the cells were digested by 0.25% trypsin (Gibco BRL, USA) and seeded at a 9-well plates (1×10^6^ cells/well), followed by incubation at 37˚C with 5% CO_2_ for 24 hours. Eugenol was added the next day and apoptosis was detected after 24 hours, 48 hours and 72 hours respectively. For detection, the samples were digested with 0.25% trypsin and resuspended with 400 ul of 1× Binding Buffer for CK groups and 100 ul 1× Binding Buffer for treated LPS groups. Then, the samples were mixed with 5 μl FITC-Annexin V, as well as 5 μl PI. CK group were divided into non-dyeing group, FITCAnnexin V group, PI group, FITC-Annexin V and PI group. The progression of cell cycle was subsequently monitored based on flow cytometry, and the result was analyzed based on Multi-Cycle AV software (Phoenix Flow Systems, USA).

### Statistical analyses

All data were expressed as mean ± standard deviation (SD). Statistical analysis was conducted with Graphpad prism 5 (Graphpad Software, USA). Furhtermore, the P<0.05 was considered to be significantly different.

## Results

### *MYC* silencing on different loci in H9C2 cells

**Fig.1:**
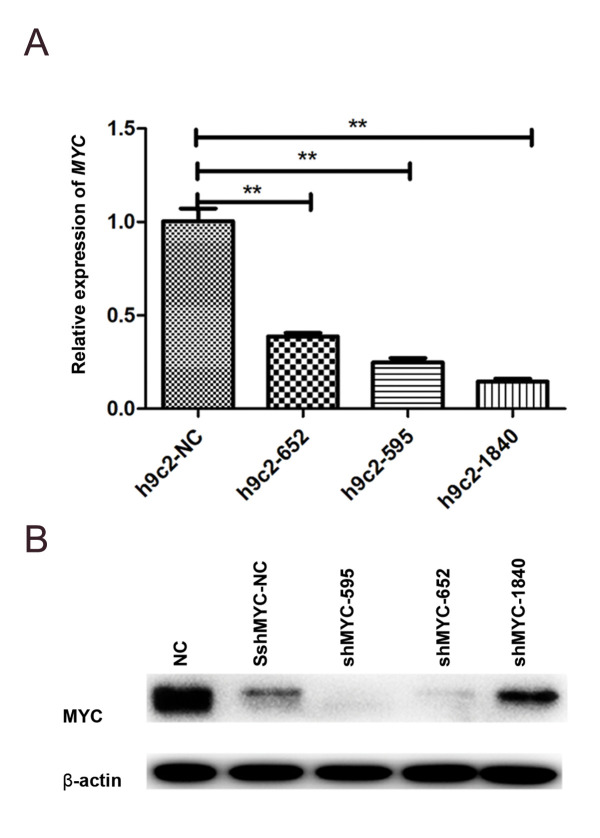
Result of *MYC* gene silencing in the current cell lines. **A.** Analysis of quantitative reverse transcription-polymerase chain reaction (qRT-PCR) to detect the effect of *MYC* silencing.** B.** Result of Western blot for protein expression in MYC silencing cells. **; P<0.01 and NC; Black control.

The effect of *MYC* gene silencing on sepsis cells had been investigated by qRT-PCR and Western blot. Compared to NC, silencing efficiency of the three loci (H9C2-652, H9C2-595 and H9C2-1840) was decreased significantly (P<0.01, Fig .1A). Rather than NC, expression of the proteins was inhibited in all *MYC* silencing samples. Meanwhile, silencing efficiency of shMYC-595 was significant, compared to the other two loci (Fig .1B).

### *MYC* silencing inhibited H9C2 cell proliferation

**Fig.2:**
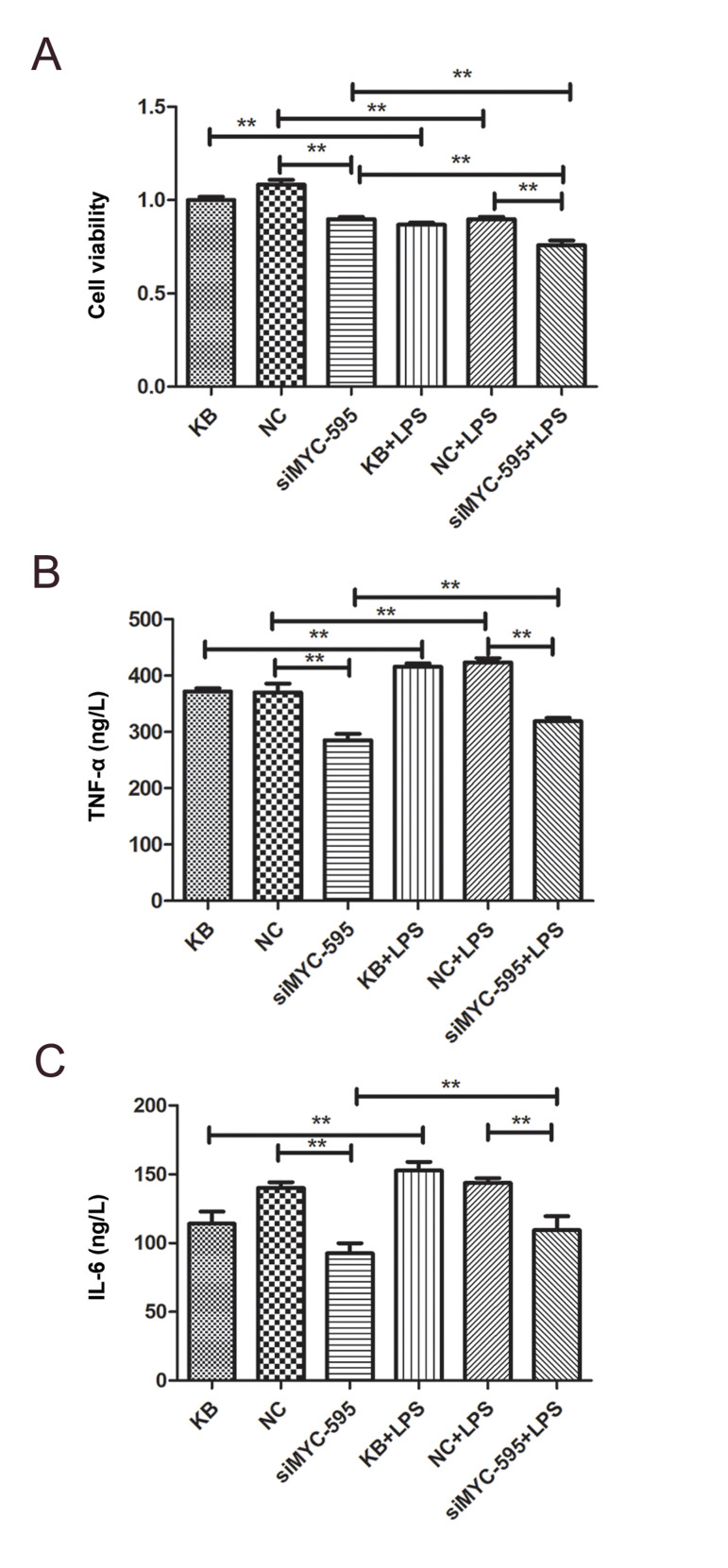
Result of CCK-8 assay and ELISA analysis. **A.** Detection of cell proliferation by CCK-8 assay.** B.** Content of TNF-α in the samples detected by ELISA. **C.** Content of IL-6 in the samples detected by ELISA. **; P<0.01 and NC; Black control.

Cell proliferation in different groups was analyzed by CCK-8 assay (Fig .2A). The result showed that compared to NC group, *MYC* silencing significantly inhibited proliferation of H9C2 cells. After LPS treatment, *MYC* silencing continued to inhibit proliferation of H9C2 cells. Moreover, in comparison with untreated cells, LPS treatment inhibited proliferation of H9C2 cells (P<0.01).

### *MYC* silencing regulated expressions of TNF-α and IL-6

The contents of TNF-α and IL-6 in H9C2 cells were analyzed by ELISA assay. The results showed that expressions of TNF-α and IL-6 were significantly decreased in *MYC* silencing group, compared to the NC group (P<0.01 for the both molecules; Fig.2B). After LPS treatment, expressions of TNF-α and IL-6 were significantly increased in *MYC* silencing group (P<0.01, Fig .2C).

### *MYC* silencing contributed to the apoptosis of H9C2 cells

**Fig.3:**
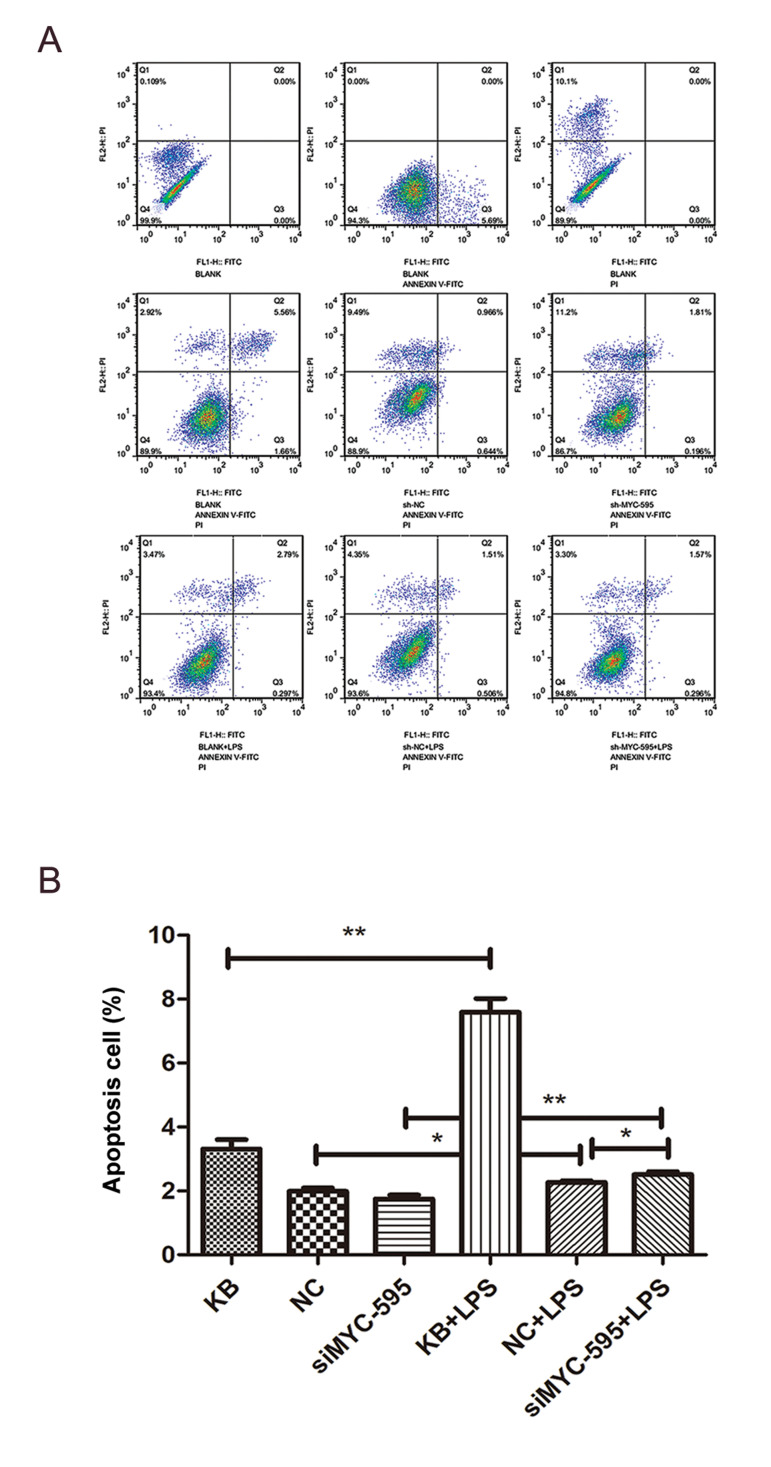
Result of flow cytometry assay for apoptosis. **A.** Result of twodimensional scatter plot for cell apoptosis in each group. PI and annexin V were represented in X-axis and Y-axis, respectively. PI-stained cells represented dead cells, annexin V-stained cells represented early apoptotic cells, and double-stained cells represented middle apoptotic cells. **B.** Apoptosis rate in different groups. PI; Propidium iodine, *; P<0.05, **; P<0.01, and NC; Black control.

The result showed that compared to the non-LPS treatment groups (KB, NC and siMYC-595), the ratio of apoptosis of H9C2 cells in LPS treatment groups (KB+LPS, NC+LPS and siMYC-595+LPS) were increased (P<0.05, Fig .3A). Moreover, compared to the NC+LPS group, apoptosis in the siMYC-595+LPS group was increased (P<0.05, Fig .3B).

### Inflammation factors analysis

**Fig.4:**
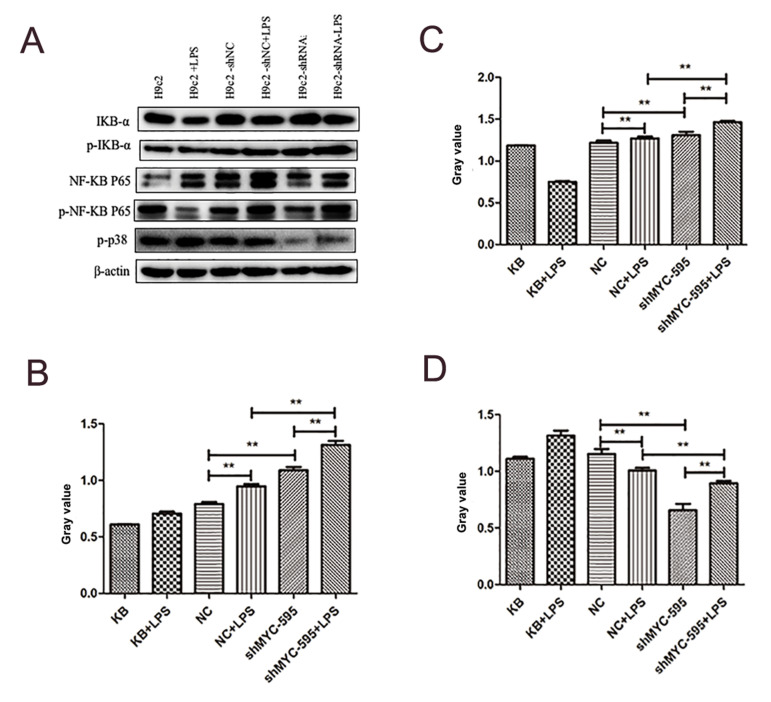
Result of Western blot for detection of inflammation factors. **A.** Electrophoresis results for some inflammation factors including pIKB-α, p-NF-KB P65 and p-P38 in different groups. **B-D.** Expression of some factors including pIKB-α, p-NF-KB P65 and p-P38 in different groups. **; P<0.01 and NC; Black control.

The inflammation factors in cells from different groups were investigated by Western blot (Fig .4A). The level of p-IKB-a in the cells treated with LPS was obviously higher than that in the untreated cells (P<0.01, Fig .4B). Meanwhile, the expression level of p-IKB-a was significantly higher in *MYC* group, in comparison with the NC group (P<0.01). Moreover, in the treated LPS group, the level of p-NFKB P65 in NC and siMYC group was observably higher than that in the non-LPS treatment group (P<0.01, Fig .4C). Compared to the NC group, expression level of p-NFKB P65 was significantly higher in silent *MYC* group (P<0.01). Meanwhile, the expression level of p-P38 was significantly higher in both siMYC and KB group in comparison to non-treated LPS group (P<0.01, Fig .4D). Furthermore, compared to the NC group, expression level of p-P38 was significantly lower in siMYC group (P<0.01).

## Discussion

The incidence of sepsis still remains high worldwide (12). Although genes such as *MYC* are thought to be associated with sepsis (10), detail of the mechanism of these genes on sepsis progression is yet unclear. This study explored the potential mechanism of *MYC* in sepsis. The result of CCK-8 assay showed that silencing *MYC* significantly inhibited cellular proliferation cells with or without LPS treatment. ELISA assay showed that expressions of TNF-α and IL-6 were decreased in *MYC* silencing group, but increased after LPS treatment. Moreover, flow cytometry assay showed that *MYC* silencing contributed to the apoptosis of sepsis cells. Furthermore, the expression of inflammatory factors investigated by Western blot showed that *MYC* silencing elevated the expression of p-IKB-a and p-NF-KB P65.

*MYC* proteins are key regulators of mammalian cell proliferation (13). A previous study showed that overexpression of *MYC* gene contributed to the cell proliferation during development of renal clear cell carcinoma (14). Developmental vascular regression is regulated by *MYC* associated pathway that controls cell proliferation (15). Wang et al. (16) indicated that *MYC* participated in the proliferation of breast cancer by promoting the gene expression including SNHG12. Moreover, the function of *MYC* in cell proliferation can be verified by *MYC* gene silencing. For example, Nayak et al. revealed that the down-regulation of *MYC* led to the tumor cell growth in human gastric cancer (17). The *MYC* silencing suppressed the interleukin-1β-induced rat chondrocyte cell proliferation and cytokine expression (18). In the hepatocellular carcinoma, RNAi silencing of *MYC* is proved to inhibit not only migration but also proliferation (19). In the current study, CCK-8 assay showed that *MYC* silencing inhibited proliferation in LPS induced sepsis cells. Thus, we speculated that *MYC* might contribute to the cell proliferation in LPS-induced sepsis.

Actually, deregulated expression of *MYC* not only promotes proliferation, but also can either induce or sensitize cells to apoptosis (20). A previous study showed that *MYC* regulates epithelial cell proliferation and control apoptosis in a positive autocrine feedback loop (21). *MYC* can induce apoptosis in various diseases such as liver and colorectal cancers (22, 23). However, flow cytometry assay in this study showed that silencing *MYC* promoted apoptosis in sepsis cells. This result indicated that *MYC* might suppress but not induce apoptosis within progression of disease.

Sepsis is caused by an inflammatory immune response triggered by an infection (9). Previous study demonstrated an important role of *MYC* in inflammatory phenotype, further indicating the vital physiological function of *MYC* in the process of inflammation (24). Due to the association with inflammation, *MYC* is a critical prognostic factor in the development of hepatoma carcinoma cell (25). A recent study shows that by programming inflammation and immune suppression, the acute activation of high levels of *MYC* can induce cellular proliferation (26). In chronic liver disease, *MYC* play a vital role in the development of disease via interacting with mediators of inflammation (27). In this study, Western blot analysis showed that deregulation of inflammation factors induced by LPS was enhanced by *MYC* silencing. Thus, we speculated that *MYC* might reduce inflammation during the progression of LPS induced sepsis. However, there were some limitations in the current study such as small simple size and lack of verification analysis. Thus, further verification studies based on the large sample size are needed to confirm all speculations in this study.

## Conclusion

*MYC* might take part in the process of LPS induced sepsis via promoting cell proliferation and inhibiting cell apoptosis. Moreover, *MYC* might reduce inflammation during the progression of LPS induced sepsis.
